# High oleic peanuts improve parameters leading to fatty liver development and change the microbiota in mice intestine

**DOI:** 10.29219/fnr.v64.4278

**Published:** 2020-08-28

**Authors:** Elise Taieb Bimro, Ran Hovav, Abraham Nyska, Tal Assa Glazer, Zecharia Madar

**Affiliations:** 1 Institute of Biochemistry, Food Science and Nutrition, Robert H. Smith Faculty of Agriculture, Food and Environment, The Hebrew University of Jerusalem, Rehovot, Israel; 2 Department of Field Crops and Vegetables Research, Plant Sciences Institute, Agricultural Research Organization, Bet-Dagan, Israel; 3 Toxicologic Pathology, Timrat and Sackler School of Medicine, Tel Aviv University, Tel Aviv, Israel

**Keywords:** high oleic peanut, liver, lipids, triglycerides, microbiota

## Abstract

**Background:**

Oleic-acid consumption can possibly prevent or delay metabolic diseases. In Israel, a Virginia-type peanut cultivar with a high content of oleic acid has been developed.

**Objective:**

This study examined the effect of consuming high oleic peanuts (D7) on the development of fatty liver compared to the standard HN strain.

**Design:**

The two peanut cultivars were added to normal diet (ND) and high-fat (HF) mouse diet. Male C57BL/6 mice were fed for 8 and 10 weeks on a 4% D7, 4% HN, or control diet. At the end of the experiments, blood and tissues were collected. Triglyceride, lipid levels, histology, and protein expression were examined. The diets’ effects on intestinal microbiota were also evaluated.

**Results:**

Both D7 and HFD7 led to a reduction in plasma triglycerides. Lipids, triglycerides, and free fatty acids in the liver were low in diets containing D7. Additionally, CD36 expression decreased in the D7 group. Consumption of D7 led to higher *Prevotella* levels, and consumption of ND that contained HN or D7 led to a lower Firmicutes/Bacteroidetes ratio.

**Conclusion:**

These findings suggest that consumption of peanuts high in oleic acid (D7) may have the potential to delay primary fatty liver symptoms.

## Popular scientific summary

- The effect of D7 on the development of fatty liver compared to the standard HN strain was examined.
- The addition of high oleic peanuts led to a decrease in the accumulation of lipids, triglycerides, and free fatty acids in the liver.
- Adding peanuts rich in oleic acid to a standard diet and a high-fat diet helps creating a more host-friendly microbiota.

Peanuts originated in Central America and were subsequently distributed to other regions of the world (1). Today, peanuts are among the most important legume crops in the world and are also considered as oilseeds due to their high fat content. In addition to oil, a wide range of peanut products, such as peanut butter, flour, milk, and more, have been developed (2).

Peanuts contain about 50% monounsaturated fatty acids (MUFAs), 33% polyunsaturated fatty acids (PUFAs), and 14% saturated fatty acids. Most of the fatty acids found in peanuts are in the form of triglycerides, which account for 93.3% to 95.5% of the total fatty acid weight. The standard peanut varieties have an oil profile containing about 52% oleic acid and about 27% linoleic acid. Roasted peanuts contain about 21.5% carbohydrates with starch being the main carbohydrate (3). Peanuts are considered to have a low glycemic index (4), and consuming peanuts or peanut oil is associated with a reduced risk of cardiovascular disease and may improve the lipid profile (5–7). High consumption of peanuts or peanut butter was shown to protect against the development of diabetes (8). Peanut butter and even peanut oil, when combined with a weight loss diet, enables the maintenance of stable long-term body weight (9). Despite these positive effects of peanut consumption, their effect on fatty liver disease has hardly been studied.

Non-alcoholic fatty liver disease (NAFLD) is the most common liver disease in the Western world, with disease incidence reaching 25–30% of the total population (10). Fatty liver disease is a continuum of liver damage, ranging from fat storage in steatosis to steatohepatitis, with or without tissue scarring. The risk of NAFLD proportionally increases with the number of metabolic syndrome clinical features (11). Fatty liver disease is influenced by both genetic and environmental factors. The central risk factors of NAFLD are identical to those defined for metabolic syndrome risk: abdominal obesity, type 2 diabetes, dyslipidemia, and insulin resistance, especially when the disease progresses (10). However, the pathogenesis of the disease is still not fully known. One major theory of the disease development is that insulin resistance causes a delay in lipolysis in fat tissue. This leads to an increased export of free fatty acids from the fat tissue to the liver, which are stored in liver cells, a condition that induces the development of fatty liver (12).

The current worldwide tendency is to shift from ‘regular’ peanut cultivars, which usually have a ratio of 1.5:2 oleic/linoleic acids in the mature seed, to high oleic acid peanuts, reaching a ratio of 15:1 (about 80% oleic acid and only about 4% linoleic acid) (13). The first high oleic acid peanut cultivar was developed in Florida by a spontaneous mutation found on the Spanish-type line (14). In Israel, Virginia-type cultivars with high oleic acid content have been developed in recent years. The fatty acid profile in these varieties is about 80% oleic acid, which is almost identical to that of olive oil. Oleic acid is almost all the MUFA fat intake in the diet (about 93%). In obese and type 2 diabetes rodents, high oleic peanut consumption led to anti-diabetic and anti-inflammatory effects. Peanuts rich in oleic acid have been found to increase insulin production and improve blood glucose measurements (15). Additionally, consuming peanuts rich in oleic acid significantly leads to more moderate glycemic responses and decreases insulin concentration and post-meal TNFα inflammation (16). Peanuts rich in oleic acid (56 g/day) given to people for 12 weeks showed that despite an increased energy uptake, their bodyweight remained unchanged but their lipid profile improved relative to a normal diet (17). Consumption of peanuts rich in oleic acid was recently shown to enhance cognitive activity (18). However, there is almost no work focused on the effect of consuming peanuts rich in oleic acid on liver health and on the development of fatty liver disease.

Interactions exist between the intestines and the liver. This ‘gut-liver axis’ plays a central role in maintaining health (19). Thus, when the GI barrier is damaged, the liver is exposed to toxic factors, and conversely, physiological disorders of the liver can cause intestinal dysfunctions. There are increasing numbers of studies identifying intestinal microbiota as environmental factors that affect obesity and the development of fatty liver disease. Studies done on nut consumption have shown that it leads to ‘friendly’ microbiota development (20). However, these studies did not include peanuts. Therefore, it is worthwhile to evaluate the effect of peanuts, especially those with high oleic acid content on the intestinal microbiota.

We hypothesize that consuming high oleic acid content peanuts will have additional benefits that will result in health improvements such as low-fat accumulation in the liver and improved lipid profile. The general aim of the present study was to examine the effect of peanuts with high oleic acid content on the development of fatty liver. This effect includes liver fat accumulation, liver enzyme levels, inflammation measures, and insulin resistance. We also examined the composition of the gut bacterial population in relation to the metabolic effects mentioned.

## Materials and methods

### Experimental animals, diets, and sample collection

All experiments were performed within the guidelines of the Authority for Biological and Biomedical Models and were approved by the Animal Care Ethics Committee, both of the Hebrew University of Jerusalem (AG-16-14784-2). Two experiments were conducted.

Experiment 1: The diets were based on the standard diet (normal diet – ND).

In the first experiment, 27 mice (male C57BL/6J mice from Harlan Laboratories, Jerusalem, Israel), aged 6–7 weeks with a mean weight of 21 ± 0.2 g, were randomly assigned to three groups, nine animals in each group, and housed in four different cages. After 4 days of acclimation, the mice were fed for 8 weeks the following diets: Standard diet (normal diet – ND), standard diet plus 4% (w/w) peanuts from cv. Hanoch (HN), and a standard diet plus 4% (w/w) peanuts from cv. D7.

Experiment 2: The diets were based on the high-fat diet – HF (excluding the control group – ND)

For the second experiment, 36 mice aged 5–6 weeks and weighing 18.3 ± 0.2 g were randomly assigned to four groups, nine animals in each group. Each group was fed the following diets for 10 weeks: normal diet (ND), high-fat diet (HF), high–fat diet plus 4% w/w Hanoch peanut (HFHN), and high-fat diet plus 4% (w/w) D7 peanut (HFD7). Nakamura and Terauchi noted that a fat-rich diet is widely used in animal steatosis production. However, various levels of steatosis and inflammation are obtained depending on the model animal, dietary fat composition, and duration of treatment (21).

The mice and food intake were measured weekly. The mice were housed in a controlled environment (12/12 h light/dark cycle, 18–24°C) with *ad libitum* access to food and water. Both HN and D7 are Virginia marketing-type extra-large seeded varieties (1.3–1.4 g). HN is a leading variety for the in-shell industry in Israel. It is a spreading type, late-maturing cultivar, and it is a ‘regular’ oleic-acid type. D7 (“Einat”) is the first registered high-oleic Virginia-type peanut cultivar in Israel, which was released by the Agricultural Research Organization. It is bunch type, middle maturing cultivar. The seed composition of both varieties is presented in Table 1. The standard diet was based on the AIN-93G diet with slight changes (see Table 2).

**Table 1:** Seed composition of the two peanut varieties

Ingredients	Experiment 1			Experiment 2		
	Normal diet	HN	D7	High-fat diet	HFNH	HFD7
Casein	19.7	19.7	19.7	27	27	27
L-Methionine	0.18	0.18	0.18	0.18	0.18	0.18
Corn starch	39.32	39.32	39.32	16.07	16.07	16.07
Dextrose	14.5	14.5	14.5	6.5	6.5	6.5
Sucrose	9.5	9.5	9.5	4.5	4.5	4.5
Cellulose	5	5	5	5	5	5
Soybean oil	7.05	7.05	7.05	25.4	25.4	25.4
Palm oil	0	0	0	10.6	10.6	10.6
Peanut	0	4	4	0	4	4
Mineral mix	3.5	3.5	3.5	3.5	3.5	3.5
Vitamin mix	1	1	1	1	1	1
Choline chloride	0.25	0.25	0.25	0.25	0.25	0.25
BHT	0.014	0.014	0.014	0.014	0.014	0.014
Total	100	104	104	100	104	104
Protein (%)	20	19	19.5	20	19.6	19.7
Carbohydrate (% Kcal)	64	61	61	20	19.7	19.7
Fat (%)	16	20	19.5	60	60.7	60.6
Total Kcal for 100 g	396.3	406.7	405.4	541	568	566

HN – Hanoch, regular peanut strain; D7 – high oleic peanut strain; HFHN – high-fat diet plus 4% (w/w) of HN; HFD7 – high-fat diet plus 4% (w/w) of D7.

**Table 2:** Composition of diets that were applied in the two experiments

Compounds	Peanut variety	
	Hanoch (HN)	Einat (D7)
Carbohydrates %	18.70	19.50
Protein %	20.00	22.50
Fat %	57.10	53.50
C16:0 (palmitic acid) %	5.59	5.75
C18:0 (stearic acid) %	2.72	2.52
C18:1n9 cis (oleic acid) %	54.63	79.89
C18:1n9 trans (trans oleic acid) %	0.46	0.75
C18:2n6 (linoleic acid) %	24.98	2.85

HN – Hanoch, the regular peanut strain; D7 – the high oleic peanut strain.

### Oral glucose tolerance test (OGTT)

A glucose-loading test was performed on weeks six and seven of the first and second experiment, respectively. Prior to the OGTT, the mice were weighed and marked and were given D-glucose (3 g/kg body weight) by gavage. Glucose levels were monitored at 0, 30, 60, and 120 min after the glucose loading. A glucometer was used to measure glucose levels in the blood drawn from the tail tip.

### Animal sacrifice and organ collection

At the end of the experiment, the mice were fasted for 12 h, their body weights were recorded, and then sacrificed by isoflurane. Blood was collected from the vena cava, and plasma was obtained by centrifugation at 5,000 g at 4°C for 10 min and stored at -20°C. Adipose tissue ans liver were collected and weighted. A small sample from the right lobe was placed in 4% formaldehyde, and the remaining liver tissue was minced in liquid nitrogen and stored at -75°C. The cecum was removed, and its contents were frozen for microbiota analysis.

### Plasma analysis

Serum lipid profiles for blood liver enzymes and a metabolic profile were performed by American Laboratories (Herzliya, Israel). Plasma insulin levels were measured by RAT/MOUSE Insulin ELISA Kit (Cat # EZRMI-13K), supplied by Merck-Millipore.

Free fatty acid levels were measured in the plasma using the Free Fatty Acid Quantification Kit (Abcam ab65341) according to the manufacturer’s instructions. The method is based on the conversion of free fatty acids to CoA derivatives when COA is oxidized. They then react with a probe to produce a color product that can be measured at 570 nm. The absolute amount of free fatty acids was calculated by a calibration curve with palmitic acid as a standard

### Liver analyses

#### Total lipid quantification

Lipid quantification in liver tissue was performed using Folch’s method. Basically, about 100 mg of liver tissue was homogenized in 700 µL of methanol. Then, 1,400 µL of chloroform was added. The samples were then mixed and left overnight at room temperature to separate the phases. The upper aqueous phase was removed, and the lower lipid phase was evaporated and weighed.

#### Composition of fatty acids in liver tissue

Approximately 200 mg of liver tissue was homogenized on ice in 2 mL of sodium chloride 0.9% solution. The fats were extracted using Folch’s method, with the addition of heptadecanoic acid (C17: 0) as an internal standard, followed by gas chromatography (GC 6890N, Agilent Technologies, Wilmington, DE).

#### Triglyceride levels

The liver triglyceride levels were measured using the Triglyceride Quantification Assay Kit (Abcam, ab-65336), according to the manufacturer’s instructions. The method is based on the process of triglycerides decomposing to free fatty acids and glycerol, while glycerol undergoes an oxidation process and reacts with a probe to produce a color product which is read at 570 nm. The absolute amount of triglycerides was calculated based on a calibration curve prepared with a standard triglyceride solution.

#### Histological examination

Histological slides were prepared by Patholab (Rehovot, Israel). The tissues were embedded in paraffin, and serial sections (3–5 µm thick) were cut from each block and stained with hematoxylin and eosin (H&E). The histopathological evaluation was performed by Prof. Abraham Nyska, a board certified toxicologic pathologist and an expert in liver histopathology. In particular, the state-of-the-art morphological criteria in the field were used (reference: https://focusontoxpath.com/articles/Proliferative-and-Nonproliferative-Lesions-of-the-Rat-and-Mouse-Hepatobiliary-System.pdf). Histopathological changes were described and scored according to a semiquantitative 0–4 scale: 0 = no lesion; 1 = minimal change; 2 = mild change; 3 = moderate change; 4 = marked change. Concurrent with histopathological evaluations, the findings in each treated animal were compared with those in a control animal to validate any putative treatment-related effects.

### Protein extraction and Western blotting

Total protein was extracted from the tissue liver with a lysis buffer containing: 20 mM Tris-HCl (pH 7.4), 145 mM NaCl, 10% glycerol, 5 mM EDTA, 1% Triton X-100, 0.5% NP-40, 100 µM PMSF, 200 µM NaVO4, 5 mM NaF, and 1% protease inhibitor cocktail. Lysates were centrifuged at 20,000 G for 15 min, and the protein concentration was determined by the Bradford method with BSA used as a standard. The samples were subjected to 7.5–10% SDS-PAGE, after which proteins were transferred onto nitrocellulose membranes. Blots were incubated with primary antibodies: anti-rabbit AMPK, pAMPK (Thr-172) (Cell Signaling Technology, Beverly, MA, USA), CD36 (Abcam, UK), and then, after several washes, with secondary goat antibodies (Jackson Immuno Research Laboratories, West Grove, PA, USA). The immune reaction was detected by enhanced chemiluminescence, with bands being quantified by densitometry and expressed as arbitrary units. An unspecific band out of the total protein (Ponceau) was used as housekeeping protein (22, 23).

### Quantitative real-time polymerase chain reaction (RT-PCR)

Total RNA was isolated from the tissue liver using Tri-Reagent (Sigma-Aldrich, Rehovot, Israel), according to the manufacturer’s protocol. Complementary DNA was prepared with the High-Capacity cDNA Reverse Transcription Kit (Quanta BioSciences, Gaithersburg, MD, USA). RT-PCR was performed with the 7300 RT-PCR System (Applied Biosystems, Foster City, CA, USA), with specific primers. Quantitative changes in gene expression were determined by normalizing against 18S mRNA.

#### Design of primers

Primer sequences were determined using the National Center for Biotechnology Information (NCBI) software. For each pair of primers, calibration was performed to determine the optimal dilution for the gene amplification (not more than five cycles from the cycle in which the S18 gene) and to ensure that no specific products or primer-dimers are produced (Table 3).

**Table 3:** Primers sequences

Name	Reverse	Forward
18S	5’-CCTCAGTTCCGAAAACCAAC-3’	5’-ACCGCAGCTAGGAATAATGG-3’
CD36	5’-AAAGGCATTGGCTGGAAGAA-3’	5’-TCCTCTGACATTTGCAGGTCTATC-3’
Fasn	5’-GGTCGTTTCTCCATTAAATTCTCAT-3’	5’-CTAGAAACTTTCCCAGAAATCTTCC-3’
G6pase	5’-AAGAGATGCAGGAGGACCAA-3’	5’ACTCCAGCATGTACCGGAAG-3’
iNOS	5’-TCTCTGCTCTCAGCTCCAAG-3’	5’-AGCTCCCTCCTTCTCCTTCT-3’
PEPCK	5’-TGCAGGCACTTGATGAACTC-3’	5’-CAAACCCTGCCATTGTTAAG-3’
PPARα	5’-CTGCGCATGCTCCGTG-3’	5’-CTTCCCAAAGCTCCTTCAAAAA 3’
Srebp-1c	5’-TAGATGGTGGCTGCTGAGTG-3’	5’-GATCAAAGAGGAGCCAGTGC-3’
TNFα	5’-CCACAAGCAGGAATGAGAAGA-3’	5’-ACGTGGAACTGGCAGAAGAG-3’

18S – 18S ribosomal RNA; CD36 – cluster of differentiation 36; Fasn – fatty acid synthase gene; G6pase – glucose 6-phosphatase; iNOS – I nitric oxide synthases; PEPCK – phosphoenolpyruvate carboxykinase; PPARα – peroxisome proliferator-activated receptor alpha; Srebp-1c – sterol regulatory element-binding transcription factor 1; TNFα – tumor necrosis factor alpha.

### Metagenomic

#### Preparation of 16S ribosomal RNA gene amplicons for the Illumina system

To study the effect of each diet on the gut microbiome, the prokaryotic 16S ribosomal RNA gene (16S rRNA) was analyzed. It is approximately 1,500 bp long and contains nine variable regions interspersed among conserved regions. These variable regions were subjected to phylogenetic classification according to genus or species in diverse microbial populations. The following protocol describes a two-step PCR-based method for preparing samples for sequencing the variable V3 and V4 regions of the 16S rRNA gene. Bacterial DNA was extracted from the mice with the PureLink Genomic DNA Mini Kit (Invitrogen, Paisley, UK). Each sample then was quantified with a Qubit 2.0 Fluorometer (ThermoFisher Scientific, Waltham, MA, USA) and diluted to a final concentration of 5 ng/µL in 10 mM Tris at pH 8.5. The 16S library preparation was carried out as described in Illumina’s 16S sample preparation guide with minor modifications. The PrimeStar HS DNA polymerase premix (Takara-Clontech, Mountain View, CA, USA) was used instead of the PCR enzyme.

### Statistical analysis

Values are presented as means ± SEM. Analysis of variance (one-way ANOVA) and the Tukey-Kramer HSD post-hoc test were used to compare means. The significance level was *P* < 0.05 for all analyses. The JMP 7.0.2 and JMP 11 Pro software suites (SAS Institute, Cary, NC, USA) were used for the analyses.

## Results

### The effect of peanut supplement on mice weight, food intake, and adipose and liver tissues weight

**Fig. 1:**
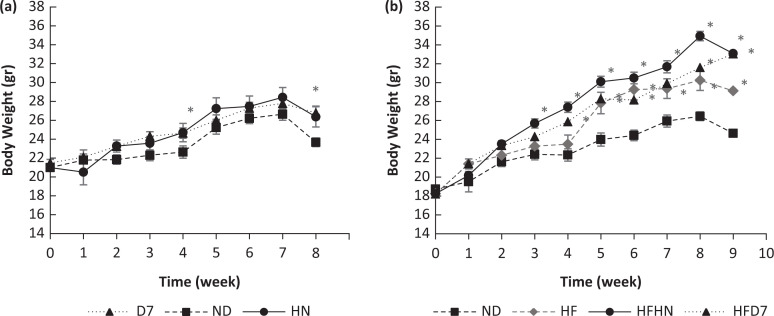
Body weight throughout the experiments. The body weight of the mice was taken every week throughout the experiments. In the first experiment (a), the mice consumed a standard diet (ND), a standard diet plus 4% (w/w) HN or D7 peanuts for 8 weeks. In the second experiment (b), the mice consumed a standard diet (ND), a high-fat diet (HF), a high-fat diet plus 4% (w/w) of HN (HFHN) or D7 (HFD7) peanuts. An ANOVA (Dunnett’s) statistical test was performed. The values presented are mean ± SE (*n* = 8–9); * represents statistical difference at *P* < 0.05 compared to the control group.

The effect of adding 4% peanuts from regular and high oleic cultivars was examined in two experiments. In the first experiment, peanuts were added to an ND, and in the second experiment, they were added to a high-fat diet. Figure 1 shows the body weight of mice fed normal (a) or high-fat (b) diets with the addition of different peanut types. Throughout the first experiment (a), no bodyweight difference was observed between the experimental groups (except week 4). At the end of the experiment, the mean weights of the HN and D7 groups were higher than in the ND group (Fig. 1a, Table 4). The D7 diet led to a significant increase in liver weight and a higher ratio of liver weight to body weight compared with other groups. However, no differences were observed in the fat tissue weight between the various treatments (Table 4, Exp 1).

**Table 4:** Initial and final body weight, food intake, and tissue weight

Parameters	Groups (Exp 1)			
	Normal diet	HN		D7
Initial body weight (g)	21.00 ± 0.09	20.99 ± 0.10		21.49 ± 0.12
Final body weight (g)	23.70 ± 0.46	26.40 ± 1.07*		26.20 ± 0.71*
Food intake (g/day)	3.30 ± 0.10	3.45 ± 0.16		3.30 ± 0.16
Liver weight (g)	0.90 ± 0.03^b^	0.96 ± 0.03^b^		1.11 ± 0.05^a^
Liver weight/ body weight (%)	3.80 ± 0.08^b^	3.63 ± 0.05^b^		4.21 ± 0.11^a^
Adipose tissue weight (g)	0.65 ± 0.06	0.79 ± 0.10		0.70 ± 0.06

Parameters	Groups (Exp 2)			
	Normal diet	High-fat diet	HFHN	HFD7

Initial body weight (g)	18.74 ± 0.36	18.24 ± 0.12	18.24 ± 0.33	18.20 ± 0.34
Final body weight (g)	24.63 ± 0.49^b^	29.10 ± 0.88^a^	33.10 ± 1.46^a^	33.05 ± 1.48^a^
Food intake (g/day)	3.10 ± 0.08^ab^	2.60 ± 0.13^b^	3.20 ± 0.20^a^	2.80 ± 0.12^ab^
Liver weight (g)	0.90 ± 0.02^b^	1.15 ± 0.06^ab^	1.33 ± 0.09^a^	1.30 ± 0.09^a^
Liver weight/ body weight (%)	3.60 ± 0.05^b^	3.90 ± 0.12^ab^	4.10 ± 0.16^a^	3.90 ± 0.14^ab^
Adipose tissue weight (g)	0.63 ± 0.02^c^	1.29 ± 0.12^b^	1.86 ± 0.17^a^	1.76 ± 0.15^ab^

HN – Hanoch, the regular peanut strain; D7 – the high oleic peanut strain; HFHN – high-fat diet plus 4% (w/w) of HN; HFD7 – high-fat diet plus 4% (w/w) of D7. Effect of diets on body weight, food intake, and tissue weight. In the first experiment (Exp. 1), the mice fed a normal diet (ND), normal diet plus 4% (w/w) peanut HN or D7 for 8 weeks. In the second experiment (Exp. 2), the mice fed a normal diet (ND), a high-fat diet (HF), a high-fat diet plus 4% (w/w), HN (HFHN), or D7 (HFD7) for 10 weeks. Tukey–Kramer and ANOVA (Dunnett’s) tests were used. Different letters indicate statistical differences, *P* < 0.05

* indicates significant differences compared to ND with *P* < 0.05.

**Fig. 2:**
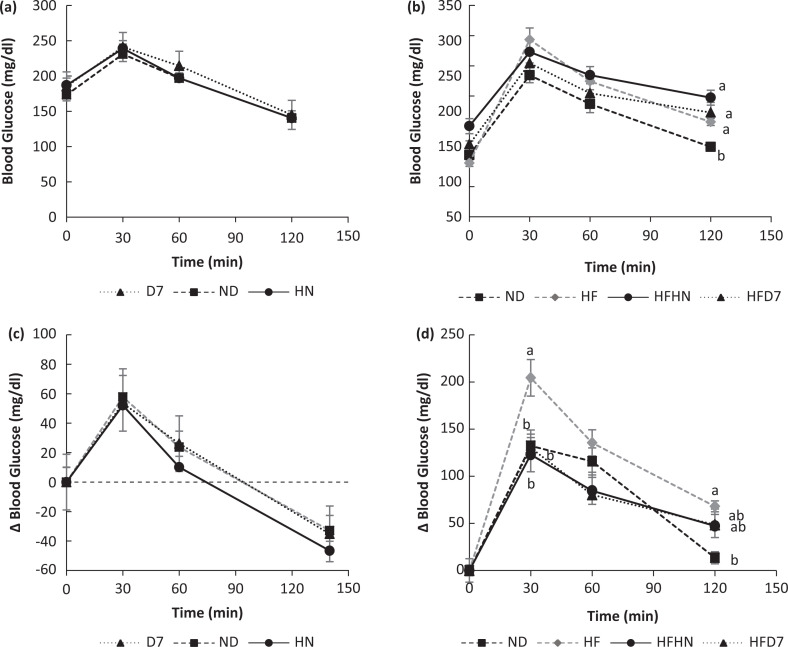
The effect of diets on glucose tolerance. The two experiments were conducted as described in Fig. 1. A week before the end of the experimental period, an oral glucose tolerance test (OGTT) (a, b) was performed. The change in glucose levels from fasting glucose levels was calculated (c, d). The mean areas under the curves (AUC) were calculated (e, f). Tukey–Kramer test was used, and the values are presented as mean ± SE, (*n* = 7–9). Different letters indicate statistical differences, *P* < 0.05.

In the second experiment, from the fifth week to the end of the experiment, the experimental group had a higher body weight compared to the control group (ND) (Fig. 1b, Table 4). When the daily food intake was measured, only an increase in HFHN versus HF was observed (Table 4, Exp. 2). The groups receiving peanut supplementation showed significantly higher liver weight than the control group (Table 4, Exp 2), and the liver weight-to-body weight ratio was highest in the HFHN group compared to the control group (Fig. 2b). HFHN showed increased fat tissue weight gain compared with HF (Table 4, Exp 2).

### The effect of diets with peanut supplement on the glycemic response

In the seventh week (Exp 1) and the ninth week (Exp 2), a OGTT was performed. In the first experiment, no differences were observed after the loading (Fig. 2a) and also no changes from fasting glucose levels (Fig. 2c). No differences were seen in the areas under the curves (AUC) between the groups (Table 5). In the second experiment, no difference was observed, but after 120 min after glucose loading, the control group (ND) showed low blood glucose levels compared to the other groups (Fig. 2b). When the results are presented as changes (Fig. 2d) in glucose levels (∆), it can be seen that after 30 min a significant difference was observed between the HF and the other experimental groups. After 120 min, high levels of glucose in the HF group were observed compared to the ND group. The area under the glucose curve was higher in the HF group, and no difference was observed between ND and the experimental groups HFHN and HFD7 (Table 5).

**Table 5:** Lipid profiles, liver enzymes, and insulin levels in plasma

Parameters	Groups (Exp 1)			
	Normal diet	HN		D7
Triglycerides (mg/dl)	104.2 ± 5.7^a^	100.1 ± 9.3^a^		67.1 ± 4.4^b^
Total Cholesterol (mg/dl)	118.6 ± 6.2	121.9 ± 5.9		125.1 ± 3.8
HDL Cholesterol (mg/dl)	106.3 ± 5.5	111.2 ± 5.6		114.0 ± 3.5
AST ( IU/L )	44.3 ± 1.4	55.0 ± 7.4		43.9 ± 3.0
ALT ( IU/L )	19.8 ± 1.0	35.0 ± 8.1		22.7 ± 3.3
ALP ( IU/L )	76.1 ± 4.0	83.7 ± 3.6		89.3 ± 4.4
Free Fatty Acid (nmol/μL)	0.23 ± 0.01	0.22 ± 0.02		0.19 ± 0.01
Insulin (ng/mL)	1.5 ± 0.23	1.9 ± 0.51		1.7 ± 0.25
AUC (mg/dl X 120 min)	3,790 ± 567	3,415 ± 532		4,194 ± 506

Parameters	Groups (Exp 2)			
	Normal diet	High-fat diet	HFHN	HFD7

Triglycerides (mg/dl)	88.0 ± 8.7	73.2 ± 7.5	69.8 ± 5.8	61.5 ± 7.6*
Total Cholesterol (mg/dl)	127.2 ± 1.9^c^	157.8 ± 4.8 ^b^	205.5 ± 6.4^a^	185.3 ± 10.4^a^
HDL Cholesterol (mg/dl)	115.1 ± 1.3^c^	143.9 ± 4.4^b^	177.9 ± 4.1^a^	163.2 ± 5.9^a^
AST ( IU/L )	55.7 ± 6.8	49.2 ± 5.8	46.8 ± 1.7	49.5 ± 3.8
ALT ( IU/L )	22.8 ± 2.3	31.3 ± 8.5	26.5 ± 2.3	27.3 ± 4.2
ALP ( IU/L )	72.0 ± 1.2	70.2 ± 4.1	70.3 ± 1.4	67.7 ± 6.0
Free Fatty Acid (nmol/μL)	0.23 ± 0.01	0.19 ± 0.01*	0.19 ± 0.01*	0. 17 ± 0.01**
Insulin (ng/mL)	1.4 ± 0.1^b^	1.9 ± 0.2^b^	3.3 ± 0.4^a^	1.8 ± 0.5^ab^
AUC (mg/dl X 120 min)	7,373 ± 787^ab^	10,183 ± 860^a^	6,071 ± 1,804^b^	6,968 ± 1,706^ab^

HN – Hanoch, the regular peanut strain; D7 – the high oleic peanut strain; HFHN – high-fat diet plus 4% (w/w) of HN; HFD7 – high-fat diet plus 4% (w/w) of D7. Effect of diets on lipid profiles, liver enzymes, and insulin levels in plasma. In the first experiment (Exp. 1), the mice fed a normal diet (ND), normal diet plus 4% (w/w) peanut HN or D7 for 8 weeks. In the second experiment (Exp. 2), the mice fed a normal diet (ND), a high-fat diet (HF), a high-fat diet plus 4% (w/w), HN (HFHN), or D7 (HFD7). At the end of both experiments, plasma triglycerides, cholesterol and HDL-cholesterol (high-density lipoprotein – cholesterol), alkaline phosphatase (AST), glutamic-pyruvic transaminase (ALT), glutamic oxaloacetic transaminase (ALP), free fatty acid levels, and insulin levels were measured in plasma and the mean areas under the curves (AUC) (*n* = 6–9). Values are mean ± SE. Tukey–Kramer and ANOVA (Dunnett’s) tests were used. Different letters indicate statistical differences, *P* < 0.05

* indicates significant differences compared to ND with *P* < 0.05, and

** indicates significant differences compared to ND with *P* < 0.001.

### The effect of diets supplemented with peanuts on plasma lipid profile, free fatty acid levels, liver enzymes, and insulin levels

In the first experiment, the D7 diet led to a significant reduction in triglyceride levels but there were no significant differences in cholesterol levels between the diet groups. Levels of plasma liver enzymes AST, ALT, and ALP were not statistically significant in experimental groups. There were no differences in the levels of free fatty acids in the plasma but the D7 group had a tendency to decrease as did the fasting plasma insulin levels at the end of the experiment (Table 5, Exp 1). In the second experiment, the triglyceride levels were lower in HFD7 than in ND, while no significant differences were found in the other groups. However, there was a significant increase in total cholesterol in the HFHN and HFD7 groups compared to HF and ND, with higher cholesterol levels in HF. A similar trend was also observed in HDL-cholesterol levels. The different diets did not affect the levels of the various liver enzymes (AST, ALT, ALP). In the HFD7 group, a decrease in plasma fatty acid levels was observed in comparison to the ND control group. Fasting plasma insulin levels at the end of the experiment were higher in HFHN than in the ND and HF group (Table 5, Exp 2).

### The effect of diets enriched with peanuts on lipid accumulation in liver tissue

**Fig. 3:**
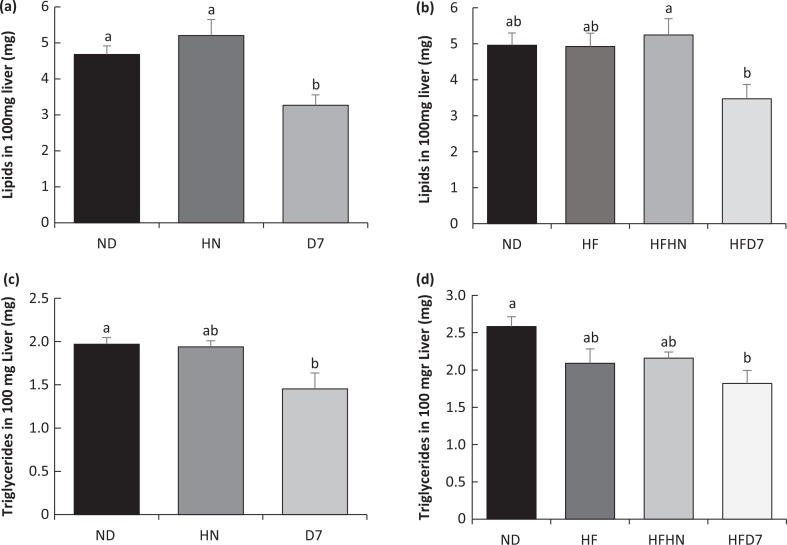
The effect of diets on lipid accumulation in the liver. In the first experiment (Exp 1), the mice consumed a standard diet (ND), a standard diet plus 4% (w/w) HN or D7. In the second experiment (Exp 2), the mice consumed a standard diet (ND), a high-fat diet (HF), a high-fat diet plus 4% (w/w) HN (HFHN), or D7 (HFD7). The fat accumulation in the liver tissue (a, b, for Exp 1 and Exp 2, respectively) and triglycerides were measured in 100 mg tissue (c, d for Exp 1 and Exp 2, respectively). A Tukey–Kramer post-hoc test was carried out. Different letters indicate statistical differences of *P* < 0.05; (*n* = 6–8).

**Fig. 4:**
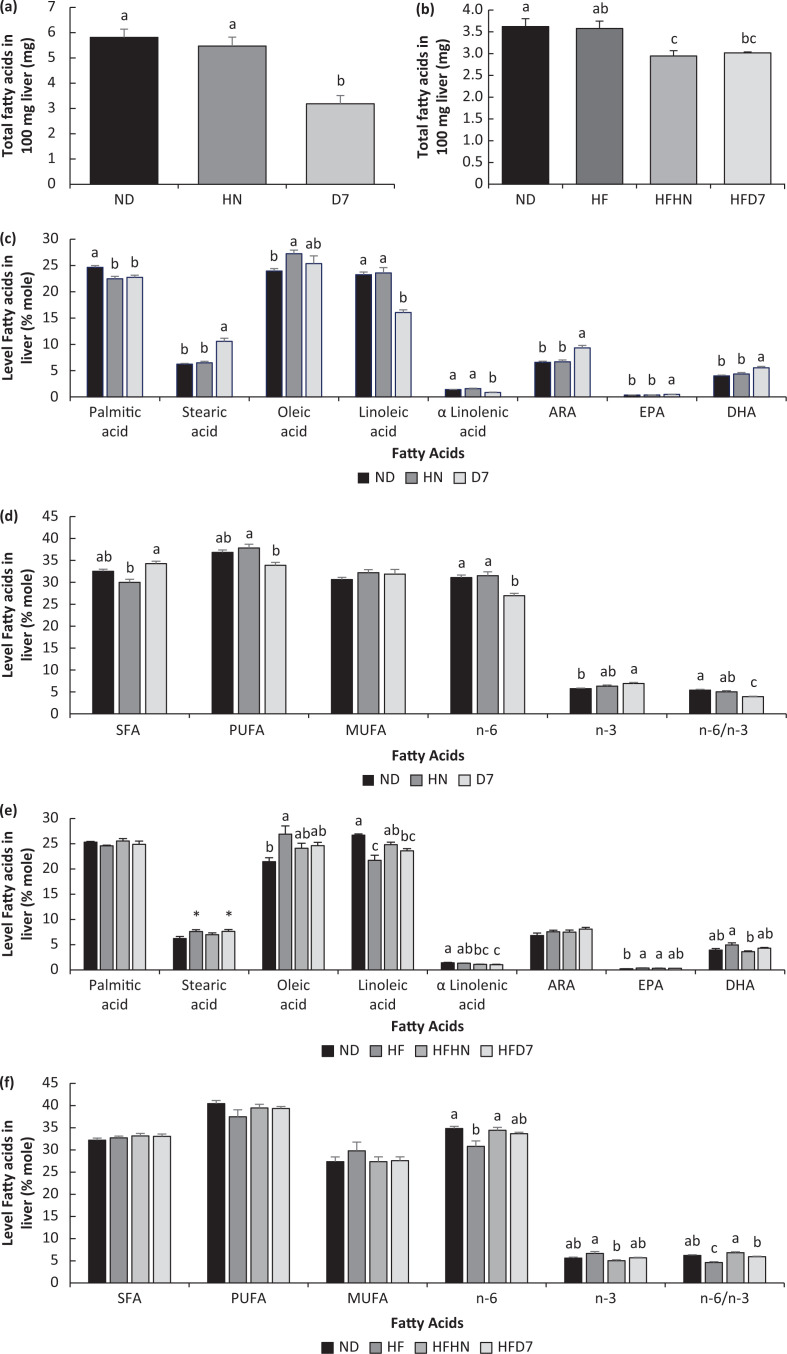
Levels of fatty acids in the liver tissue. In the first experiment (Exp 1), the mice consumed a standard diet (ND), a standard diet plus 4% (w/w) HN or D7. In the second experiment (Exp 2), the mice consumed a standard diet (ND), a high-fat diet (HF), a high-fat diet plus 4% (w/w), HN (HFHN) or D7 (HFD7). The total level of free fatty acids was measured in 100 mg of tissue. The molecular concentration of fatty acids (c17 was used as an internal standard) and the molecular concentration of different types of fatty acids in liver tissue are described in Fig. 4a, c and e for experiment 1 and in Fig. 4b, d and f for the second experiment. A Tukey–Kramer post-hoc test was carried out. Different letters indicate statistical differences, *n* = 5; *P* < 0.05.

As shown in Fig. 4a, there was a significant decrease in the percentage of fat accumulation in the liver of mice consuming the D7 diets compared with ND and HN. Triglyceride levels in the D7 group were significantly lower than in the ND group (Fig. 3c). Total FFA levels in the liver were lower in D7 compared to HN and ND (Fig. 4a). The FFA levels palmitic acid (C16:0) was significantly lower in the HN and D7 groups than in the ND group. The ratio of stearic acid (C18:0), arachidonic acid (C20:4 n-6), eicosapentaenoic acid (EPA, C20:5n3), and docosahexaenoic acid (DHA, C22:6 n-3) was higher in D7 than in the other groups (Fig. 4c). We also saw low levels of linoleic acid (C18:2 n-6 cis 9,12) and α linoleic acid (C18:3 n-3 cis 9,12,15) in the D7 group compared to other groups. Between the ND and D7 levels, there was no difference in the oleic acid (C18:1 n-9 cis) levels, whereas in the HN group, this acid level was high compared to ND and D7 (Fig. 4c). In Exp. 2, there was a significant decrease in the percentage of fat accumulation in the liver of mice consuming the HFD7 diets compared with ND and HFHN (Fig. 3b). A similar trend was found with triglycerides level (Fig. 3d).

Regarding the total of free fatty acids levels in the liver tissue, HFD7 and HFHN were found to lead to lower fatty acid levels in the liver compared to ND, especially in the group that consumed the HFHN diet (Fig. 4b). The levels of oleic acid (C18:1 n-9 cis) and EPA (C20: 5n3) were lower in the ND group compared to the HF group. ND tended to increase the levels of linoleic acid (C18:2 n-6 cis9,12) and α linoleic acid (C18: 3n3 cis 9,12,15) compared with all diets except HFHN and HF, respectively. The addition of D7 to ND increased saturated fatty acid (SFA) levels, while PUFAs were lower in the D7 group, but no differences in MUFAs levels were found between the experimental groups. Omega 6 was lower in the D7 group and omega 3 was higher. This resulted in a lower omega 6/omega 3 ratio in the D7 group (Fig. 4e). In the HF experiment, there were no differences between groups in the levels of all fatty acids. The omega 6/omega 3 ratio was low in the HF and D7 groups compared to the HN group (Fig. 4f).

**Fig. 5:**
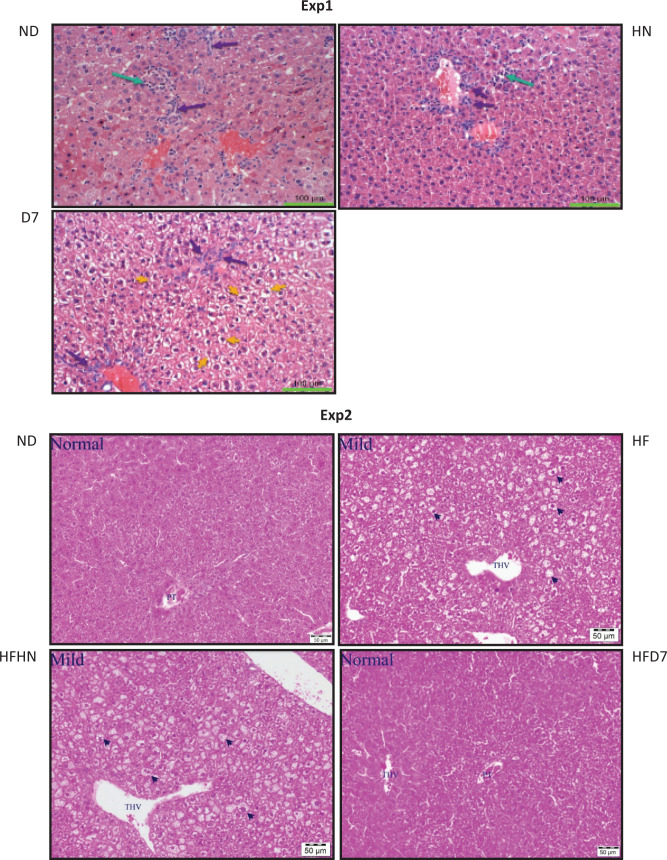
Liver tissue histology. The mice consumed a standard diet (ND), standard diet plus 4% (w/w) HN or D7 peanut varieties (Exp 1) for 8 weeks. In the second experiment, the mice consumed a standard diet (ND), a high-fat diet (HF), a high-fat diet plus 4% (w/w), HN (HFHN) or D7 (HFD7) peanut varieties. After the mice were sacrificed, pieces of the liver tissue were removed and stained with H&E. A representative image of liver histology (enlarged X200) is demonstrated.

The morphological and histological changes in the liver are described in Fig. 5. In the liver samples from all groups, minimal grades of inflammatory cell infiltration, with or without oval cell proliferation were noted. These kinds of lesions, of similar severity, are known to occur as background changes. Hepatocytic cytoplasmic pallor, reflecting glycogen accumulation, was noted only in the D7 group (all animals). Moreover, the yellow arrows in the representative photos of liver from Group D7 are indicating the presence of mixed micro- and macro-vesicular hepatocytic vacuolation. Fatty vacuolation can be reversible, without further damage to the liver.

The morphological and histologic changes in the liver (Fig. 5, Exp 2) show that in the ND group, it is not apparent that the phenomenon of micro- and macro-vesicular steatosis developed, while the HF led to minimal steatosis. The HFHN diet has led to the progressive development of steatosis. Supplementation of peanuts HFD7 led to mild micro- and macro-vesicular steatosis signs but all signs of fatty vacuolation can be reversible (24) (Fig. 5 Exp 2).

### The effect of diets enriched with peanuts on carbohydrate metabolism in the liver

**Fig. 6:**
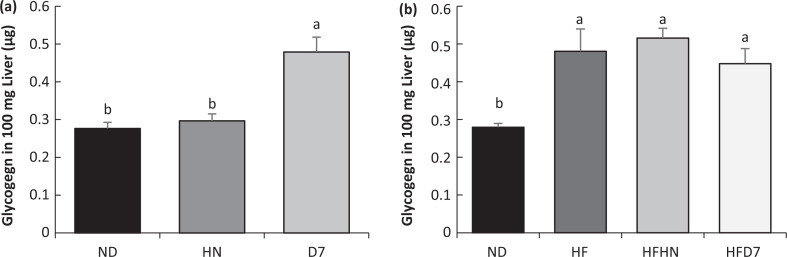
The effect of diets on the glycogen level in the liver. In the first experiment (a), the mice consumed a standard diet (ND), standard diet plus 4% (w/w) HN or D7 peanuts for 8 weeks. In the second experiment (b), the mice consumed a standard diet (ND), a high-fat diet (HF), a high-fat diet plus 4% (w/w) of HN (HFHN) or D7 peanuts (HFD7) for 10 weeks. The amount of glycogen in the liver tissue was expressed per 100 mg of tissue. An ANOVA (Dunnett’s) statistical test was performed. The values presented are mean ± SE (*n* = 8–9). A Tukey–Kramer post-hoc test was carried out. Different letters indicate statistical differences, *P* < 0.05.

**Fig. 7:**
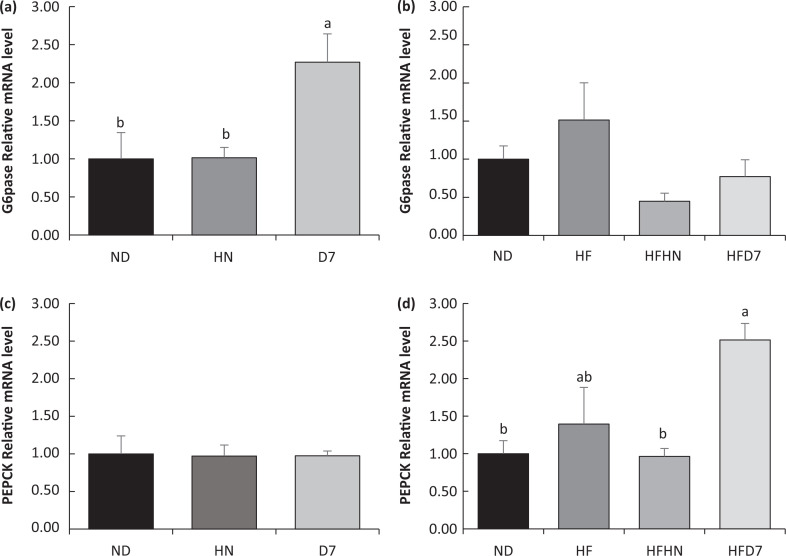
Effect of diets on gene expression involved in gluconeogenesis in the liver tissue. In the first experiment (Exp. 1), the mice consumed a standard diet (ND), a standard diet plus 4% (w/w) HN or D7 for 8 weeks. In the second experiment (Exp. 2), the mice consumed a standard diet (ND), a high-fat diet (HF), a high-fat diet plus 4% (w/w), HF (HFHN) or D7 peanuts (HFD7). G6pase (a, b) and PEPCK (c, d) mRNA levels were measured using RT-PCR. The results were normalized to the 18S gene expression. A Tukey–Kramer post-hoc test was carried out. Different letters indicate statistical differences at *P* < 0.05; (*n* = 6–8).

To assess the dietary effect on carbohydrate metabolism, glycogen levels were determined in the liver tissue (Fig. 6). The expression levels of key enzymes involved in gluconeogenesis were also examined at both the mRNA and protein levels (Fig. 7). In the first experiment, glycogen levels were higher in the D7 diet compared to the other experimental groups (Fig. 6a). These results correspond to the histological results (Fig. 5). In the second experiment (Fig. 6b), glycogen levels were lower in the control group (ND) compared to the other groups.

G6pase, which plays a significant role in gluconeogenesis and glycogen hydrolysis, had elevated mRNA levels in the livers of the D7 mice (Fig. 7a). No similar effect was seen in either HFHN or HFD7 diets (Fig. 7b). The expression level of mRNA-PEPCK remained similar in all ND groups with and without peanuts (Fig. 7c). The HFD7 diet led to an increase in PEPKK mRNA compared to other diets (Fig. 7d).

### The effect of peanuts consumption on gene and protein expression contributing to fat metabolism in the liver

**Fig. 8:**
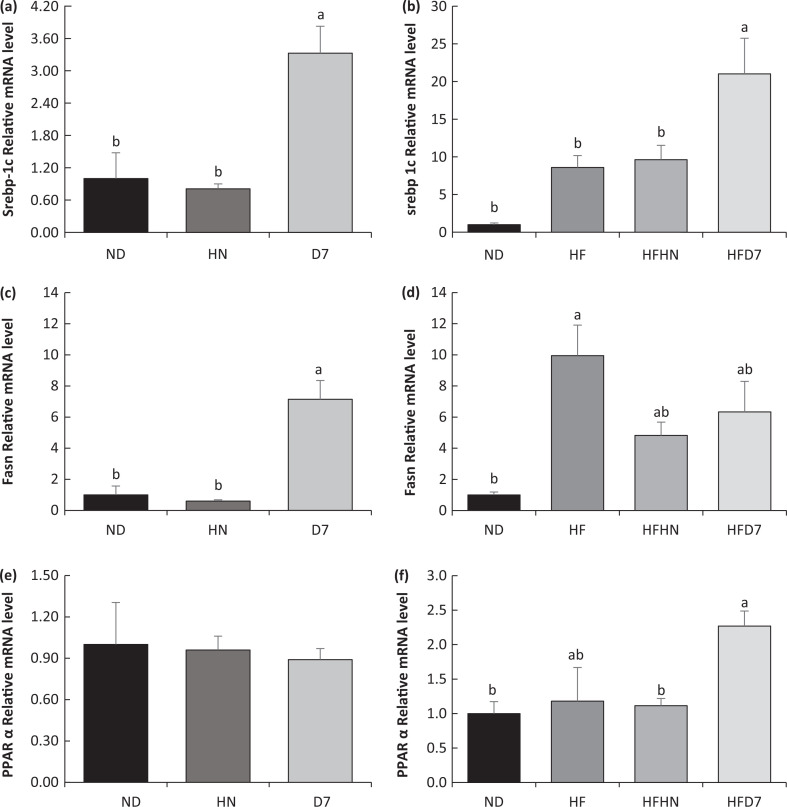
Effect of diets on gene expression involved in fat metabolism in the liver tissue. In the first experiment (Exp. 1), the mice consumed a standard diet (ND), a standard diet plus 4% (w/w) of HN or D7 for 8 weeks. In the second experiment (Exp. 2), the mice consumed a standard diet (ND), a high-fat diet (HF), a high-fat diet plus 4% (w/w), HN peanuts (HFHN) or D7 (HFD7). Expression of Srebp-1c, Fasn, and PPARα gene was measured using RT-PCR. The results were normalized to 18S gene expression. A Tukey–Kramer post-hoc test was carried out. Different letters indicate statistical differences, *P* < 0.05; (*n* = 6–8).

To examine the effect of peanuts on fat metabolism in the liver tissue, Srebp-1c, PPARα, and Fasn were selected for mRNA expression. D7 together with a standard diet led to a significant increase in the level of both Srebp-1c and Fasn genes (Fig. 8a, c). When the mice were fed a high-fat diet with D7, only the Srebp-1c gene level increased (Fig. 8b). With regard to PPARα gene expression, only mice consuming the HFD7 diet showed an increase (Fig. 8e, 8f).

**Fig. 9:**
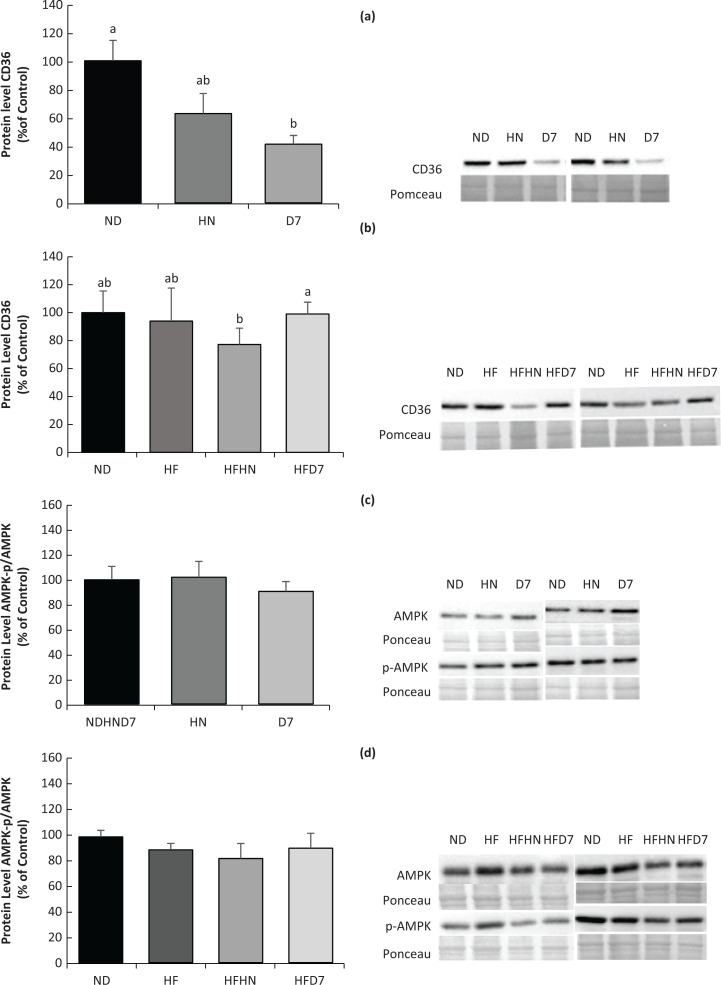
Effect of diets on protein expression involved in fat metabolism in the liver tissue. In the first experiment (Exp. 1), the mice consumed a standard diet (ND), a standard diet plus 4% (w/w) of HN or D7 for 8 weeks. In the second experiment, the mice consumed a standard diet (ND), a high-fat diet (HF), a high-fat diet plus 4% (w/w), HN (HFHN) or D7 (HFD7) peanut varieties. The protein level expressions of CD36 (Fig. 9a, b) and AMPK-p/AMPK (Fig. 9c, d) were measured using Western blot where an unspecific band out of the total protein (Ponceau) was used as a protein control. A Tukey–Kramer post-hoc test was carried out. Different letters indicate statistical differences, *P* < 0.05; (*n* = 6–8).

The CD36 protein mediates the entry of long fatty acids into liver cells and other tissues. CD36 protein levels decreased significantly in the D7 group compared with the rest of the experimental group (Fig. 9a). In the second experiment, CD36 levels in the liver increased in the HFD7 experimental group compared with HFHN (Fig. 9b). It should be noted that other gene and protein expressions such as AMPK (Fig. 9c, d) and ACC (data not shown) were measured, but no effect was observed in either experiments.

### Contribution of peanut cultivars to preventing liver inflammation development

**Fig. 10:**
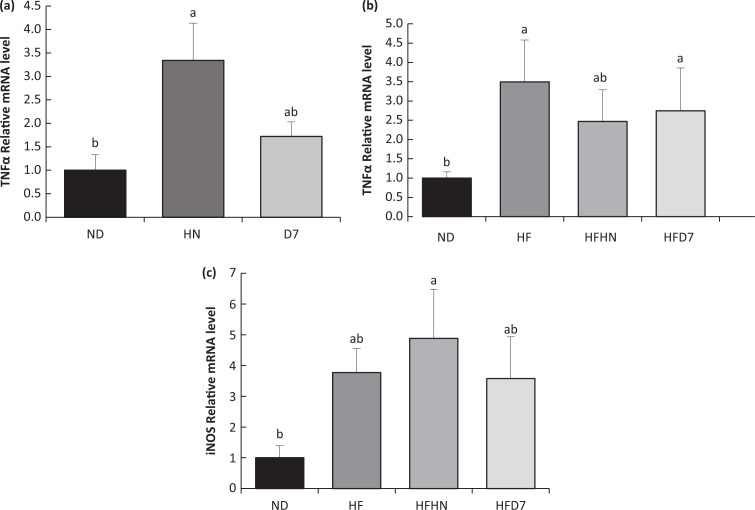
Effects of diets on liver tissue inflammation. In the first experiment (Exp. 1), the mice consumed a standard diet (ND), standard diet plus 4% (w/w) HN or D7 for 8 weeks. In the second experiment (Exp. 2), the mice consumed a high-fat diet (HF), a high-fat diet plus 4% (w/w), HN (HFHN) or D7 (HFD7) peanuts. The expression of TNFα (a, b) and iNOS gene (c) was measured at the transcription level (RT-PCR), and the results normalized to the 18S gene expression. A Tukey–Kramer post-hoc test was performed. The values displayed are mean ± standard error. Columns marked with different letters indicate statistically significant variances at *P* < 0.05; (*n* = 6–8).

To examine changes in inflammation levels following peanut consumption, the TNFα gene expression levels, an important signaling protein in inflammatory mechanisms, was measured. In the first experiment, HN led to an increase in TNFα, while D7 tended to lower TNFα expression (Fig. 10a). In the second experiment, in which the mice consumed a high-fat diet, TNFα expression increased, while HFHN tended to reduce the effect of the high-fat diet (Fig. 10b). Since we did not expect the iNOS gene expression, which is a known indicator of inflammation, to be not altered after consuming a standard diet, the effect of peanuts on the iNOS gene was examined only after a high-fat diet consumption. As shown, all diets demonstrated a high level of iNOS compared to ND, with HFD7 having a relatively lower expression compared to HF or HFNH (Fig. 10c).

### The effect of diets on the intestinal microbiota composition

**Fig. 11:**
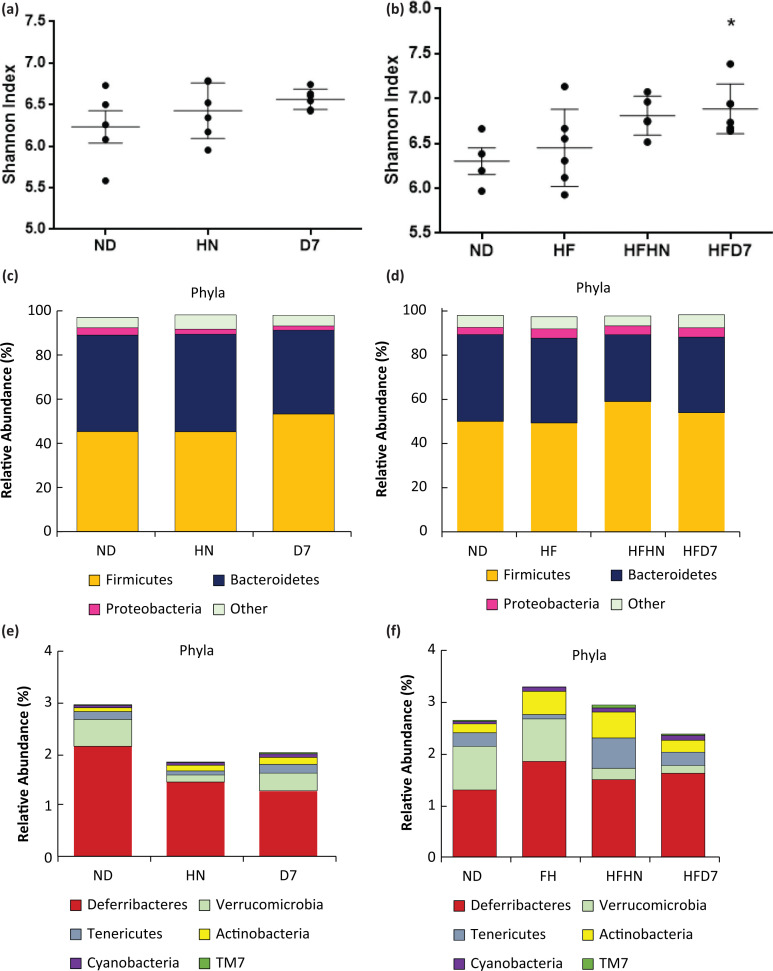
The effect of diets on the microbiota composition in the intestines. In the first experiment (Exp. 1), the mice consumed a standard diet (ND), standard diet plus 4% (w/w) HN or D7 peanuts for 8 weeks. In the second experiment (Exp. 2), the mice consumed a standard diet (ND), a high-fat diet (HF), a high-fat diet plus 4% (w/w), HN (HFHN) or D7 (HFD7) peanuts. α-Diversity with Shannon test parameters was calculated (a, b). The bacterial population was determined at the level of phyla (c, d, e, f) (*n* = 5) using S18. In graph b, an ANOVA (Dunnett’s) statistical test was performed. The values shown are averages ± standard error; * – represents statistical variance of *P* < 0.05 compared to ND.

**Fig. 12:**
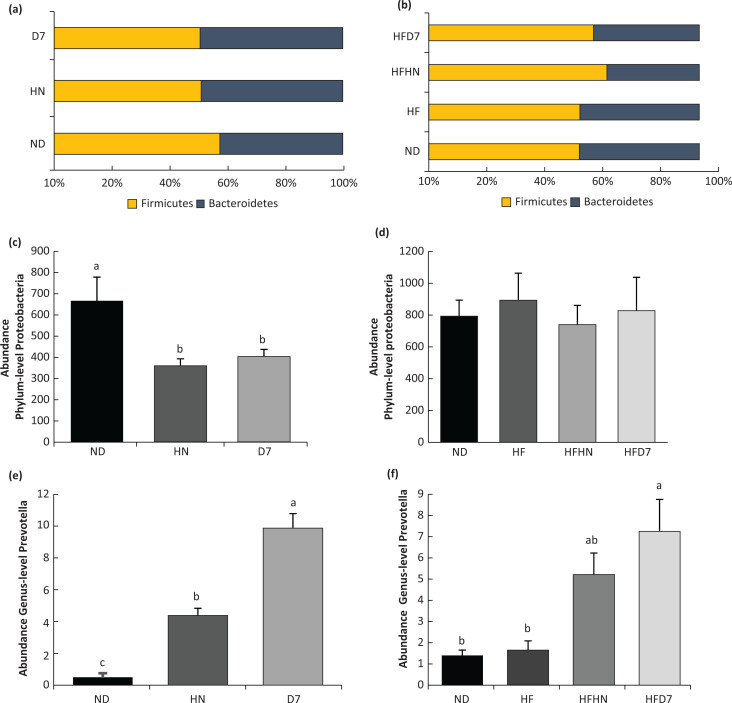
Effect of diets on the ratio of Firmicutes/Bacteroidetes and levels of Proteobacteria and Prevotella in the intestines. In the first experiment (Exp. 1), the mice consumed a standard diet (ND), standard diet plus 4% (w/w) HN or D7 peanuts for 8 weeks. In the second experiment (Exp. 2), the mice consumed a standard diet (ND), a high-fat diet (HF), a high-fat diet plus 4% (w/w), HN (HFHN) or D7 (HFD7) varieties. The ratio Firmicutes/Bacteroidetes (a, b), proteobacteria (c, d), and Prevetolla (e, f) levels (*n* = 5). The graphs present values ± standard error, and a Tukey–Kramer post-hoc test was performed at *P* < 0.05.

To examine the effect of peanuts on the intestinal bacteria population, a metagenomics analysis was performed. The Shannon test, a measure of the α-diversity of the intestinal bacterial population, was similar in all experimental groups that were fed with the ND (Fig. 11a). However, in the HF experiment, an increase in the diversity of bacteria was observed in the D7 (HFD7) group (Fig. 11b). At the phyla level, the proportion of Firmicutes was higher in the D7 ND group, and Bacteroidetes had lower levels compared to the other groups. Proteobacteria were lower in HN and D7 than in ND (Fig. 12c). Levels of Deferribateres were lower in both the HN and D7 groups. Verrucomicrobia levels were lower in groups that received additional peanuts but more significantly in the HN group (Fig. 11c, e). The ratio of Firmicutes/Bacteroidetes was lower in the HN and D7 groups compared with ND (Fig. 12a). High levels of Prevotella were seen in group D7 compared to ND, and D7 showed a difference even in comparison to the HN group (Fig. 12e). HF led to a higher proportion of Firmicutes following consumption of peanuts, significantly in the HFHN group where the percentage of Bacteroidetes was lower in the same group. Proteobacteria levels were the same for all experimental groups (Fig. 12d). Higher Deferribateres levels were found in the HF group. Verrucomicrobia were low in all the peanut supplemented groups. The Tenericutes levels were higher in the regular diet group with the addition of HN and lower in the groups consuming the HF diet (Fig. 11d, f). The ratio of Firmicutes/Bacteroidetes was higher in groups that consumed the fat-rich diets with peanut supplementation but more conspicuously in the HFHN group (Fig. 12b). At the genus level, Prevotella levels were higher in the HFD7 group compared to ND and HF (Fig. 12f).

## Discussion

Some of the peanut’s health benefits are attributed to their nutritional composition, especially to their lipid profile. The fat content in conventional varieties is about 50% MUFA and about 25% PUFA. Nutritional recommendations attach great importance to consuming up to 20% of total daily caloric intake from MUFA oils such as olive oil. The contribution of the protein that came from the peanuts to the diet in our experiments is 0.8% which is small. Therefore, we do not appear to have a significant impact on the results. However, greater protein supplementation can affect carbohydrate and fat metabolism including NAFLD. Rosario Martínez et al. showed such an effect using legume (lentil) (25). The unique fatty profile in the recently developed high-oleic varieties (D7) is similar to the olive oil profile. Few studies have examined the effect of peanut consumption with high oleic acid content on fat and carbohydrate metabolism. In this work, 4% (w/w) HN or D7 peanut cultivar species was added to the ND diet or to the HF diet. The addition of D7 or HN to an ND diet led to similar final weights, with a trend toward higher weight without differences in daily food intake (Fig. 1a, Table 4). Therefore, differences in body weight should not be related to food consumption. Mice consuming HFHN showed a trend of weight gain that was also accompanied by increased fat tissue compared to other groups (Fig. 1b, Table 4). These results can be attributed to higher dietary intake in the HFHN group. Therefore, the addition of D7, peanuts rich in oleic acids to a standard diet does not prevent weight gain and does not have an advantage over standard peanuts. These results contradict Mattes et al.’s findings, which show that daily peanut supplementation does not cause weight gain and can also help in weight loss (9). However, our HF results support the findings of Duarte Moreira Alves et al., who reported that consumption of high oleic acid content peanuts reduces the risk of obesity in humans (16).

Mice consuming either ND or HF supplemented by D7 showed an increasing trend in RNA expression of the genes involved in liver gluconeogenesis (PEPECK and G6pase, Fig. 7). These findings indicate the onset of gluconeogenesis. However, there were no differences in the level of adiponectin, AdipoR2 receptor expression, and p-AMPK expression (Fig. 9c, d) which take part in the activation of gluconeogenesis genes (26). Therefore, it is difficult to show that the gluconeogenesis amplification is due to the consumption of the peanuts rich in oleic acid.

Mice consuming HFHN showed an increase in fat tissue (Table 4), which was associated with increased adiponectin levels and an increase in plasma insulin levels (Table 5). It is also interesting to note the decrease in genes involved in gluconeogenesis (PEPCK and G6pase) in the liver (Fig. 7), but here too, without changes in p-AMPK protein levels (Fig. 9c, d). These events appear to indicate an increase in insulin sensitivity in the HFHN group, despite the increase in fat tissue weight. Choi et al. described similar processes in which adiponectin is involved via PPARγ regulation in adipose tissue (27). Our finding showing insulin secretion after peanuts consumption is supported by other studies (15). Diets supplemented with D7 led to elevated G6pase gene expression, which plays a role in the breakdown of glycogen in the liver. However, this was not reflected in our work because it was precisely the accumulation of liver glycogen in group D7 (Fig. 5 Exp. 1) that indicates liver health. Storage of glycogen in the liver is associated with an increase in liver insulin sensitivity (28). In this work, the contribution of p-AMPK protein to mediate carbohydrate and fat metabolism in the liver (29) required further elucidation.

The addition of D7 to a standard diet led to low plasma triglyceride levels (Table 5 Exp 1). Etherton et al. also reported that a MUFA-rich diet consumption led to lower triglyceride levels than the control group (ND). However, Etherton et al. also reported low cholesterol levels (7) which we did not see. Furthermore, the addition of D7 to a high-fat diet also led to low plasma triglyceride levels compared with ND (Table 5 Exp 2). These results are inconsistent with Buettner et al.’s finding that there was no differences in plasma triglyceride levels between a high-fat diet and one with olive oil (30). Plasma free fatty acid levels tended to decrease in the D7 group compared to the ND and decrease in the HFD7 group (Table 5). These findings suggest that peanuts rich in oleic acid regardless of diet can lower free fatty acids in the blood. Furthermore, Bates et al. showed that administering oleic acid into the rat bloodstream led to a reduction in the free fatty acids in the blood. These results were explained by the lipolytic activity and subsequent reduction in fat tissue (31). We hypothesize that the addition of peanuts rich in oleic acid (D7) contributes to a decrease in free fatty acids in the blood and reduces the risk of cardiovascular disease (32). However, these results do not confirm the results obtained by Buettner et al., which showed a significant increase in the levels of free fatty acids in the group consuming a high-fat diet supplemented with olive oil (30).

Mice on a standard diet with D7 showed lower liver lipid levels (Fig. 3a). Also, triglycerides and free fatty acids in the liver were lower in this group (Figs. 3c, 5a). However, unexpectedly, the liver weight in this group was higher than in the other groups (Table 4 Exp 1). This may be due to an increase in the accumulation of glycogen in the liver tissue (Fig. 6a). Interestingly, the histological outcome showed no differences in fat accumulation in the liver (Fig. 5). In mice fed HFD7, the level of steatosis was more moderate compared to HFHN (Fig. 5 Exp 2). These findings are supported by the liver lipid levels and triglycerides in the blood that were lower in HFD7 groups (Fig. 3b,d, Table 5 Exp 2) and by the relative decrease in the ratio of liver weight to body weight, again compared with HFHN (Table 4). However, liver weight was higher in both HFHN and HFD7 (Table 4 Exp 2). These results do not support the work of Cintra et al. that found lower liver weight in a group that consumed a high fat plus peanuts with no difference in lipid levels (33). However, Hussein et al. showed that MUFA derived from olive oil and incorporated into a diet-induced NAFLD reduces the accumulation of triglycerides in the liver (34). Our findings suggest that the addition of peanuts rich in oleic acid to a standard diet or a high-fat diet could lead to decreases in the accumulation of lipids in liver cells. The results obtained at the genotype level partially support the resulting phenotype. The addition of D7 to a standard diet showed a decrease in the accumulation of liver lipids (Fig. 3a) and paradoxically an increase in mRNA levels of Srebp1c and Fasn (Fig. 8a, c) which are central to the formation of fatty acids and triglycerides in the liver. At the same time, a decrease in the expression of the transporter CD36 was found (Fig. 9a). CD36 is responsible for transporting free fatty acids to the liver. It may be that the expression of Srebp-1c and Fasn indicate the possibility of compensation in group D7 in order to balance the fatty acid levels in the cell. The steatosis level was low in HFD7 compared to HFHN (Fig. 5 Exp 2). An upward trend in the expression of the Srebp-1c and PPARα genes in the HFD7 group was also found (Fig. 8d, f). These findings suggest effective lipid utilization in groups consuming oleic acid-rich peanuts, without an increase in lipid accumulation in liver tissue compared with HFHN. It is possible that adding peanuts to the diet, especially peanuts rich in oleic acid, is capable of preserving the balance between lipogenesis and lipolysis through the expression of proteins involved, thus enabling a more appropriate equilibrium between fat production and oxidation.

Analysis of liver fatty acids showed a higher content of palmitic, stearic, oleic, linoleic, and arachidonic acids (Fig. 4c, d). These findings are consistent with the results obtained in previous studies of both mice (35) and humans (36). Kawano claims that PUFA fatty acids are the most significant contributors to triglycerides (37). Our findings when adding D7 to ND support the hypothesis that a decrease in linoleic acid (PUFA) in group D7 (Fig. 5b) was consistent with a reduction in the amount of triglycerides in the liver (Fig. 4b). This finding was also seen in the HFD7 group which showed a tendency of decreases in linoleic acid levels compared to HFHN (Fig. 5f). The standard diet containing D7 led to an increase in stearic acid levels in the liver (Fig. 4c), an acid that is at the beginning of the *de novo* fatty chain synthesis. Also, the percentage of saturated fatty acids was highest in this group compared to the HN groups (Fig. 5c). We assume that these findings indicate an increase in lipogenesis in the D7 group, which was also seen in the expression of the lipogenic genes Srebp1c and Fasn (Fig. 8a, c). However, this is not consistent with fat accumulation in the D7 group’s liver. This finding requires further clarification. Djohan et al. showed that a diet rich in oleic acid from olive oil lowers the levels of PUFA fatty acids in the liver (38), which supports the results obtained in our work. In our work, adding peanuts rich in oleic acid to either diet reduced the n-6/n-3 ratio (Fig. 4e, f), suggesting an anti-inflammatory effect in the liver (35). In humans, the n-6/n-3 ratio is increased in steatosis, especially in the case of NASH (36). Therefore, consuming peanuts rich in oleic acid may reduce the risk of NAFLD by improving the tissue’s composition of fatty acids, lowering the ratio of n-6/n-3.

Among the goals of this work was to examine the effect of peanuts rich in oleic acid on the first signs of fatty liver development. Indeed, a decrease in markers for this symptom, such as low levels of lipids, triglycerides, and fatty acids in the liver (Figs. 3, 5a,b), as well as triglycerides and free fatty acids in the blood, was seen after D7 consumption (Table 5, Fig. 4). This may also prevent fibrosis development in the liver (39). This hypothesis is supported by a recent clinical study showing that a Mediterranean diet rich in virgin olive oil and/or nuts reduces the incidence of steatosis and liver fat levels after the experimental period (40). The histological findings and inflammatory gene expression levels in the D7 groups indicate that inflammation did not develop (Fig. 5). The decrease in liver lipid, triglyceride, and free fatty acids levels after D7 consumption (Figs. 3, 5a, b) can indicate that the addition of oleic acid-rich peanuts protects against fat accumulation in liver tissue, a major marker of fatty liver pathogenesis. This can also be seen by the decline in insulin levels and free fatty acids in plasma in the HFD7 group (Table 5). Therefore, peanuts rich in oleic acid can suppress the primary mechanisms of fatty liver disease manifested as decreased fat accumulation in the liver, decreased plasma fatty acid flow followed by improved insulin sensitivity.

We also considered the peanuts’ effect on the composition of the intestinal microbiota. We found that at the phyla level, the population of Bacteriodetes and Firmicutes are the most dominant in the intestine (Fig. 12c, d). Bäckhed et al. reported the same findings in humans and mice (41). In addition, a positive association was found between the ratio of Firmicutes/Bacteroidetes and BMI (42), which led to using this ratio as a potential obesity phenotype in mice and humans. The Firmicutes/Bacteroidetes ratio was lower in groups that consumed peanuts regardless of the strain (Fig. 12a, b). In addition to changes of Bacteriodetes and Firmicutes populations, there was an apparent decrease in the level of Proteobacteria in the peanut consuming groups (Fig. 12c). It has been reported that levels of Proteobacteria are lower in healthy humans and that high levels of Proteobacteria are potential indicators for dysbiosis and disease risk (43). Obesity and/or NASH showed an increase in Proteobacteria (44). Therefore, adding peanuts in a regular diet may promote and preserve a healthier microbiota. When examining the peanuts’ effect at the genus level, we see that the oleic acid-rich cultivar led to elevated Preototella levels compared with ND (Fig. 12e, f). Kovatcheva-Datchary et al. reported that Prevotella links to stimulation of glycogen accumulation in the liver and thus protects against glucose intolerance (45). These findings may explain the increase in glycogen levels in the D7 mice liver. Haro et al. also reported higher levels of Prevotella in healthy people than those with metabolic syndrome and obesity (46). In the HFD7 groups, we found an increase in the alpha-diversity according to the Shannon test (Fig. 11b), indicating an increase in the wealth and distribution of the intestinal bacterial population in these groups. A similar result was reported by Nakanishi et al. (47). In addition, Sobhonslidsuk et al. reported a downward trend in the alpha-diversity indices in subjects with NASH compared with healthy ones (48). An increase in alpha-diversity is a marker of a more friendly microbiota. We therefore suggest that adding peanuts rich in oleic acid to any diet has the advantage of developing ‘healthy’ gut microbiota.

## Conclusion

The study’s main findings show that the addition of peanuts with a high oleic acid content led to a decrease in the accumulation of lipids, triglycerides, and free fatty acids in the liver. This finding is accompanied by histological findings supporting these results and by showing less fat accumulation in the HFD7 groups than the HFHN group. We believe that low levels of liver lipids resulted from a decrease in CD36 transporter levels, which is responsible for fatty acid entry into the liver. The addition of peanuts rich in oleic acid also led to a decrease in the free fatty acid and triglyceride levels in the blood. Interestingly, the addition of oleic acid-rich peanuts to a standard diet or a high-fat diet did not show an apparent reduction in the expression of genes and proteins involved in lipogenesis. Adding oleic acid-rich peanuts to either a standard diet or high-fat diet helps create a more host-friendly microbiota and is associated with a healthier phenotype. In general, the addition of peanuts cultivar rich in oleic acid led to an increase in the wealth of the intestinal bacterial population which is linked to improvement in the metabolic syndrome.

We believe that consuming peanut rich in the oleic acid (D7) may have the potential to delay the development of the fatty liver; however, further elucidations are required.

## References

[1] Settaluri VS, Kandala CVK, Puppala N, Sundaram J Peanuts and their nutritional aspects – a review. Food Nutr Sci 2012; 3(12): 1644. doi: 10.4236/fns.2012.312215

[2] Arya SS, Salve AR, Chauhan S Peanuts as functional food: a review. J Food Sci Technol 2016; 53(1): 31–41. doi: 10.1007/s13197-015-2007-9 26787930 PMC4711439

[3] Toomer OT Nutritional chemistry of the peanut (Arachis hypogaea). Crit Rev Food Sci Nutr 2018; 58(17): 3042–53. Available from: https://www.tandfonline.com/loi/bfsn20 28662347 10.1080/10408398.2017.1339015

[4] Foster-Powell K, Holt SHA, Brand-Miller JC International table of glycemic index and glycemic load values: 2002. Am J Clin Nutr 2002; 76(1): 5–56. doi: 10.1093/ajcn/76.1.5 12081815

[5] Alper CM, Mattes RD Peanut consumption improves indices of cardiovascular disease risk in healthy adults. J Am Coll Nutr 2003; 22(2): 133–41. doi: 10.1080/07315724.2003.10719286 12672709

[6] Fraser GE, Sabate J, Beeson WL, Strahan TM A possible protective effect of nut consumption on risk of coronary heart disease: the Adventist Health Study. Arch Intern Med 1992; 152(7): 1416–24. doi: 10.1001/archinte.1992.00400190054010 1627021

[7] Kris-Etherton PM, Pearson TA, Wan Y, Hargrove RL, Moriarty K, Fishell V, et al High–monounsaturated fatty acid diets lower both plasma cholesterol and triacylglycerol concentrations. Am J Clin Nutr 1999; 70(6): 1009–15. doi: 10.1093/ajcn/70.6.1009 10584045

[8] Jiang R, Manson JE, Stampfer MJ, Liu S, Willett WC, Hu FB Nut and peanut butter consumption and risk of type 2 diabetes in women. JAMA 2002; 288(20): 2554–60. doi: 10.1001/jama.288.20.2554 12444862

[9] Mattes RD, Kris-Etherton PM, Foster GD Impact of peanuts and tree nuts on body weight and healthy weight loss in adults. J Nutr 2008; 138(9): 1741S–5S. doi: 10.1093/jn/138.9.1741S 18716179

[10] Bellentani S The epidemiology of non-alcoholic fatty liver disease. Liver Int 2017; 37: 81–4. doi: 10.1159/000282080 28052624

[11] Than NN, Newsome PN A concise review of non-alcoholic fatty liver disease. Atherosclerosis 2015; 239(1): 192–202. doi: 10.1016/j.atherosclerosis.2015.01.001 25617860

[12] Lewis GF, Carpentier A, Adeli K, Giacca A Disordered fat storage and mobilization in the pathogenesis of insulin resistance and type 2 diabetes. Endocr Rev 2002; 23(2): 201–29. doi: 10.1210/edrv.23.2.0461 11943743

[13] Isleib TG, Pattee HE, Sanders TH, Hendrix KW, Dean LO Compositional and sensory comparisons between normal-and high-oleic peanuts. J Agric Food Chem 2006; 54(5): 1759–63. doi: 10.1021/jf052353t 16506830

[14] Norden AJ, Gorbet DW, Knauft DA, Young CT Variability in oil quality among peanut genotypes in the Florida breeding program. Peanut Sci 1987; 14(1): 7–11. doi: 10.3146/i0095-3679-14-1-3

[15] Vassiliou EK, Gonzalez A, Garcia C, Tadros JH, Chakraborty G, Toney JH Oleic acid and peanut oil high in oleic acid reverse the inhibitory effect of insulin production of the inflammatory cytokine TNF-α both in vitro and in vivo systems. Lipids Health Dis 2009; 8(1): 25 http://www.lipidworld.com/content/8/1/25 19558671 10.1186/1476-511X-8-25 PMC2706835

[16] Alves RDM, Moreira APB, Macedo VS, de Cássia Gonçalves Alfenas R, Bressan J, Mattes R, et al Regular intake of high-oleic peanuts improves fat oxidation and body composition in overweight/obese men pursuing a energy-restricted diet. Obesity 2014; 22(6): 1422–9. doi: 10.1002/oby.20746. 24639419

[17] Barbour J, Howe P, Buckley J, Bryan J, Coates A Effect of 12 weeks high oleic peanut consumption on cardio-metabolic risk factors and body composition. Nutrients 2015; 7(9): 7381–98. doi: 10.3390/nu7095343 26404365 PMC4586538

[18] Barbour JA, Howe PRC, Buckley JD, Bryan J, Coates AM Cerebrovascular and cognitive benefits of high-oleic peanut consumption in healthy overweight middle-aged adults. Nutr Neurosci 2017; 20(10): 555–62. doi: 10.1080/1028415X.2016.1204744 27386745

[19] Compare D, Coccoli P, Rocco A, Nardone OM, De Maria S, Cartenì M, et al Gut–liver axis: the impact of gut microbiota on nonalcoholic fatty liver disease. Nutr Metab Cardiovasc Dis 2012; 22(6): 471–6. doi: 10.1016/j.numecd.2012.02.007 22546554

[20] Lamuel-Raventos RM, Onge M-PS Prebiotic nut compounds and human microbiota. Crit Rev Food Sci Nutr 2017; 57(14): 3154–63. doi: 10.1080/10408398.2015.1096763 27224877 PMC5646185

[21] Nakamura A, Terauchi Y Lessons from mouse models of high-fat diet-induced NAFLD. Int J Mol Sci 2013; 14(11): 21240–57. 24284392 10.3390/ijms141121240 PMC3856002

[22] Rivero-Gutiérrez B, Anzola A, Martínez-Augustin O, de Medina FS Stain-free detection as loading control alternative to Ponceau and housekeeping protein immunodetection in Western blotting. Anal Biochem 2014; 467: 1–3. 25193447 10.1016/j.ab.2014.08.027

[23] Smith NT, Soriano-Arroquia A, Goljanek-Whysall K, Jackson MJ, McDonagh B Redox responses are preserved across muscle fibres with differential susceptibility to aging. J Proteomics 2018; 177: 112–23. 29438851 10.1016/j.jprot.2018.02.015 PMC5884322

[24] Thoolen B, Maronpot RR, Harada T, Nyska A, Rousseaux C, Nolte T, et al Proliferative and nonproliferative lesions of the rat and mouse hepatobiliary system. Toxicol Pathol 2010; 38(7_suppl): 5S–81S. doi: 10.1177%2F0192623310386499 21191096 10.1177/0192623310386499

[25] Martínez R , Kapravelou G , Donaire A , et al Effects of a combined intervention with a lentil protein hydrolysate and a mixed training protocol on the lipid metabolism and hepatic markers of NAFLD in Zucker rats. Food Funct. 2018;9(2):830–850. doi: 10.1039/C7FO01790A 29364302

[26] Hardie DG, Schaffer BE, Brunet A AMPK: an energy-sensing pathway with multiple inputs and outputs. Trends Cell Biol 2016; 26(3): 190–201. doi: 10.1016/j.tcb.2015.10.013 26616193 PMC5881568

[27] Choi JS, Kim J-H, Ali MY, Min B-S, Kim G-D, Jung HA Coptis chinensis alkaloids exert anti-adipogenic activity on 3T3-L1 adipocytes by downregulating C/EBP-α and PPAR-γ. Fitoterapia 2014; 98: 199–208. 25128422 10.1016/j.fitote.2014.08.006

[28] Magnusson I, Rothman DL, Katz LD, Shulman RG, Shulman GI Increased rate of gluconeogenesis in type II diabetes mellitus. A 13C nuclear magnetic resonance study. J Clin Invest 1992; 90(4): 1323–7. 1401068 10.1172/JCI115997 PMC443176

[29] Shackelford DB, Shaw RJ The LKB1–AMPK pathway: metabolism and growth control in tumour suppression. Nat Rev Cancer 2009; 9(8): 563. 19629071 10.1038/nrc2676 PMC2756045

[30] Buettner R, Parhofer KG, Woenckhaus M, Wrede CE, Kunz-Schughart LA, Scholmerich J, et al Defining high-fat-diet rat models: metabolic and molecular effects of different fat types. J Mol Endocrinol 2006; 36(3): 485–501. 16720718 10.1677/jme.1.01909

[31] Bates MW, Linn LC, Huen AH-J Effects of oleic acid infusion on plasma free fatty acids and blood ketone bodies in the fasting rat. Metabolism 1976; 25(4): 361–73. doi: 10.1016/0026-0495(76)90068-8 1263831

[32] Engin A Non-alcoholic fatty liver disease. Adv Exp Med Biol. 2017;960:443–67. doi: 10.1007/978-3-319-48382-5_19 28585211

[33] Cintra DEC, Costa AV, Maria do Carmo GP, Matta SLP, Silva MTC, Costa NMB Lipid profile of rats fed high-fat diets based on flaxseed, peanut, trout, or chicken skin. Nutrition 2006; 22(2): 197–205. doi: 10.1016/j.nut.2005.09.003 16459232

[34] Hussein O, Grosovski M, Lasri E, Svalb S, Ravid U, Assy N Monounsaturated fat decreases hepatic lipid content in non-alcoholic fatty liver disease in rats. World J Gastroenterol 2007; 13(3): 361. 17230603 10.3748/wjg.v13.i3.361 PMC4065889

[35] da Silva-Santi L, Antunes M, Caparroz-Assef S, Carbonera F, Masi L, Curi R, et al Liver fatty acid composition and inflammation in mice fed with high-carbohydrate diet or high-fat diet. Nutrients 2016; 8(11): 682. doi: 10.3390/nu8110682 PMC5133070 27801862

[36] Yamada K, Mizukoshi E, Sunagozaka H, Arai K, Yamashita T, Takeshita Y, et al Characteristics of hepatic fatty acid compositions in patients with nonalcoholic steatohepatitis. Liver Int 2015; 35(2): 582–90. doi: 10.1111/liv.12685 25219574

[37] Kawano Y, Cohen DE Mechanisms of hepatic triglyceride accumulation in non-alcoholic fatty liver disease. J Gastroenterol 2013; 48(4): 434–41. doi: 10.1007/s00535-013-0758-5 23397118 PMC3633701

[38] Djohan YF, Badia E, Bonafos B, Fouret G, Lauret C, Dupuy A-M, et al High dietary intake of palm oils compromises glucose tolerance whereas high dietary intake of olive oil compromises liver lipid metabolism and integrity. Eur J Nutr 2019;58(8):3091–107. doi: 10.1007/s00394-018-1854-3 30392135

[39] Udomsinprasert W, Honsawek S, Poovorawan Y Adiponectin as a novel biomarker for liver fibrosis. World J Hepatol 2018; 10(10): 708. doi: 10.4254/wjh.v10.i10.708 30386464 PMC6206156

[40] Pintó X, Fanlo-maresma M, Corbella E, Corbella X, Mitjavila MT, Moreno JJ, et al A Mediterranean diet rich in extra-virgin olive oil is associated with a reduced prevalence of nonalcoholic fatty liver disease in older individuals at high cardiovascular risk. J Nutr 2019; 149(11): 1–10. doi: 10.1093/jn/nxz147 31334554

[41] Ley RE, Bäckhed F, Turnbaugh P, Lozupone CA, Knight RD, Gordon JI Obesity alters gut microbial ecology. Proc Natl Acad Sci 2005; 102(31): 11070–5. doi: 10.1073/pnas.0504978102 16033867 PMC1176910

[42] Koliada A, Syzenko G, Moseiko V, Budovska L, Puchkov K, Perederiy V, et al Association between body mass index and Firmicutes/Bacteroidetes ratio in an adult Ukrainian population. BMC Microbiol 2017; 17(1): 120. 28532414 10.1186/s12866-017-1027-1 PMC5440985

[43] Shin N-R, Whon TW, Bae J-W Proteobacteria: microbial signature of dysbiosis in gut microbiota. Trends Biotechnol 2015; 33(9): 496–503. doi: 10.1016/j.tibtech.2015.06.011 26210164

[44] Zhu L, Baker SS, Gill C, Liu W, Alkhouri R, Baker RD, et al Characterization of gut microbiomes in nonalcoholic steatohepatitis (NASH) patients: a connection between endogenous alcohol and NASH. Hepatology 2013; 57(2): 601–9. doi: 10.1002/hep.26093 23055155

[45] Kovatcheva-Datchary P, Nilsson A, Akrami R, Lee YS, De Vadder F, Arora T, et al Dietary fiber-induced improvement in glucose metabolism is associated with increased abundance of Prevotella. Cell Metab 2015; 22(6): 971–82. doi: 10.1016/j.cmet.2015.10.001 26552345

[46] Haro C, García-Carpintero S, Rangel-Zúñiga OA, Alcalá-Díaz JF, Landa BB, Clemente JC, et al Consumption of two healthy dietary patterns restored microbiota dysbiosis in obese patients with metabolic dysfunction. Mol Nutr Food Res 2017; 61(12): 1700300. doi: 10.1002/mnfr.201700300 28940737

[47] Nakanishi M, Chen Y, Qendro V, Miyamoto S, Weinstock E, Weinstock GM, et al Effects of walnut consumption on colon carcinogenesis and microbial community structure. Cancer Prev Res 2016; 9(8): 692–703. doi: 10.1158/1940-6207.CAPR-16-0026 27215566

[48] Sobhonslidsuk A, Chanprasertyothin S, Pongrujikorn T, Kaewduang P, Promson K, Petraksa S, et al The association of gut microbiota with nonalcoholic steatohepatitis in Thais. Biomed Res Int 2018; 2018: 8. doi: 10.1155/2018/9340316 PMC5842744 29682571

